# Comparative virulence studies and transcriptome analysis of *Staphylococcus aureus* strains isolated from animals

**DOI:** 10.1038/srep35442

**Published:** 2016-10-14

**Authors:** Zahid Iqbal, Mohamed N. Seleem, Hafiz Iftikhar Hussain, Lingli Huang, Haihong Hao, Zonghui Yuan

**Affiliations:** 1 MOA Laboratory for Risk Assessment of Quality and Safety of Livestock and Poultry Products, Huazhong Agricultural University, Wuhan, China; 2 Department of Comparative Pathobiology, College of Veterinary Medicine, Purdue University, West Lafayette, Indiana, USA; 3 Hubei Collaborative Innovation Center for Animal Nutrition and Feed Safety, Huazhong Agricultural University, Wuhan, China; 4 National Reference Laboratory of Veterinary Drug Residues (HZAU) and MAO Key Laboratory for Detection of Veterinary Drug Residues, Huazhong Agricultural University, Wuhan, China

## Abstract

Several studies have been conducted to check the prevalence of methicillin-resistant strains of *Staphylococcus aureus* (MRSA) in animals and animal-derived food products but limited data are available regarding their virulence and associated gene expression profile. In the present study, antibiotic resistance and virulence of MRSA and methicillin-sensitive *S. aureus* animal isolates were determined *in vitro* by agar dilution, biofilm formation, adhesion, invasion and intracellular survivability assays. In addition, the pathogenicity of these isolates was examined in a murine model of *S. aureus* sepsis. MRSA1679a, a strain isolated from chicken, was observed to be highly virulent, in cell culture and in mouse model, and exhibited extensive resistant profile. Comparative gene expression profile of MRSA1679a and the reference human MRSA strain (ATCC 29213) was performed using Illumina-based transcriptome and RT-qPCR analyses. Several virulence elements including 22 toxin genes were detected in MRSA animal-isolate. In addition, we observed enhanced expression of crucial virulence regulators, such as *sarA* and *KdpDE* in MRSA animal-isolate compared to the human isolate. Collectively, gene expression profile including several virulence and drug-resistance factors confirmed the unique and highly virulent determinants of the MRSA strain of poultry origin which warrants further attention due to significant threat to public health.

Methicillin-resistant *Staphylococcus aureus* (MRSA) is a multidrug-resistant and pathogenic bacteria causing severe community acquired and healthcare associated infections in human^1^. MRSA also causes infections in a number of animals such as lameness in poultry and mastitis in cow, leading to huge economic loss^2^. Current epidemiological studies have revealed that MRSA strains have increased in virulence posing a serious risk to public health^3^. These multidrug resistant strains have been recently detected in animal husbandry as well as in animal-derived food products raising issues of the possible zoonotic transmission^4^. Surprisingly, genetic analysis of *S. aureus* isolates from chickens, from different parts of the world, has illustrated that the majority of those isolates were most likely the result of transfer of a human isolate (ST5) to poultry^5^. The increasing prevalence of zoonotic MRSA raises a question concerning the virulence and molecular mechanisms mediating the success of these strains. While genome sequencing of MRSA isolates from animals has been identified, the gene expression profile and contribution to virulence remain unknown.

*S. aureus* has the capacity to express a large number of virulence elements and toxins which play an important role during host infection^6^. Gene expression and regulation of virulence elements in human isolates of *S. aureus* is generally governed by global virulence regulators including a staphylococcal accessory regulator (*sarA*), an accessory gene regulating system (*agrABCD*)^7^, and two component system (*KdpDE*)^8^. In addition, many virulence and resistance determinants in *S. aureus* found on mobile genetic elements (MGEs), such as staphylococcal cassette chromosomes, pathogenicity islands, plasmids, bacteriophages, transposons and insertion sequences, are also controlled by *S. aureus* global gene regulators^9^.

Little is known about the gene expression and regulation of these virulence elements in animal associated MRSA. Here, we describe the transcriptome of the clinical MRSA1679a strain derived from poultry, and compared it with human MRSA strain ATCC 29213. In addition, antimicrobial susceptibility, ability to form biofilm, adhesion and invasion, and virulence of MRSA and methicillin-sensitive *S. aureus* animal isolates were determined in pure culture, infected mammalian cell culture, and in an *in vivo* mouse model. Results garnered from this study confirm the unique virulent regulators and global gene expression profile of MRSA strain of poultry origin.

## Results

### Bacterial isolates and Antimicrobial Susceptibility Test

Two MRSA strains (478 and 1679a isolated from pig and chicken, respectively) and two methicillin-susceptible *Staphylococcus aureus* (MSSA) strains (586 and 1161a both isolated from pig) isolates were selected for this study (Supplementary Table S1). In addition, human MRSA strain ATCC 29213 was used as a reference strain^10^.

The minimum inhibitory concentrations (MICs) obtained from antimicrobial susceptibility testing for the five *S. aureus* isolates are presented in Table 1. Results had shown that strain MRSA1679a exhibits resistance to various antibiotic classes including macrolides, aminoglycosides, lincosamides, and fluoroquinolones. MRSA1679a was susceptible only to tetracycline. MRSA478 was susceptible to levofloxacin and ceftiofur while MSSA586 was susceptible to levofloxacin, oxacillin, methicillin and ceftiofur. MSSA1161a was susceptible to most of the used drugs and resistant to ampicillin, tetracycline, ceftiofur and sulfamethoxazole-trimethoprim.

### Biofilm Formation

**Figure 1:**
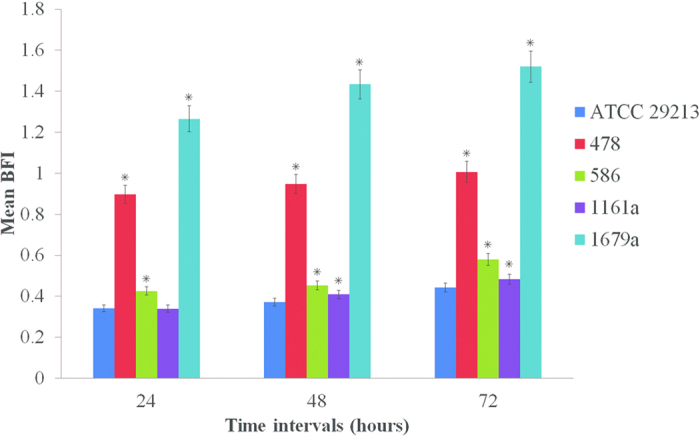
Biofilm formation of four *S. aureus* isolates at different time points. The results are presented as mean specific biofilm formation (SBF) of three independent repeats and compared to ATCC 29213. Asterisk (*) represents statistical significance (P ≤ 0.05) using two-tailed t-test.

After examining the antimicrobial susceptibility of the clinical isolates, we next investigated their ability to form biofilm *in vitro*. Different levels of biofilm formation were recorded for *S. aureus* isolates. We observed positive correlation between biofilm formation and the incubation time. The highest level of biofilm formation was observed after 72 hours of incubation. MRSA1679a was the strongest biofilm producer compared to other strains (Fig. 1). In addition, significant differences in biofilm formation were observed between MRSA strains (MRSA1679a, MRSA478) and MSSA strains (586 and 1161a).

### Adhesion, Invasion and Intracellular Survivability Assay

**Figure 2:**
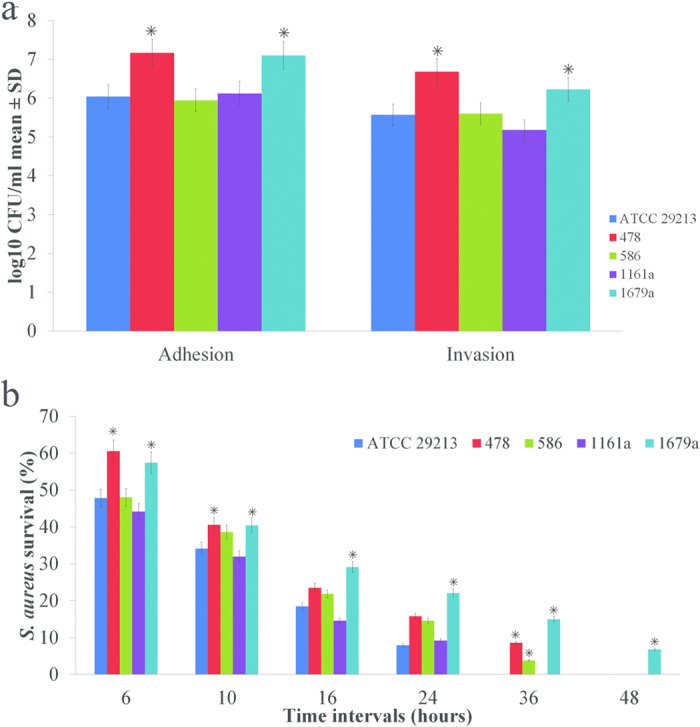
*In vitro* virulence assay of four *S. aureus* strains in macrophage RAW264.7 cells. **(a)** No. of adherent and internalized bacteria. The results are presented as log10 of the mean ± standard deviation (SD) CFU/ml of three independent repeats and compared to ATCC 29213. **(b)** intra-macrophage survival rate of *S. aureus* strains at different time intervals. The results are presented as percentage of survival rate. Asterisk (*) represents statistical significance (P ≤ 0.05) using two-tailed t-test.

Since invasion and intracellular survival of *S. aureus* constitute potent virulence components, we chose to assess the ability of *S. aureus* animal-isolates to invade and survive inside murine macrophage cell line RAW264.7. Both MSSA strains (586 and 1161a) showed a similar trend of adhesion and invasion compared to the reference strain (ATCC 29213). However, both MRSA strains from animal origin (478 and 1679a) exhibited significantly higher adhesion and invasion of the macrophage cells compared to other MSSA strains and MRSA human-reference strain (Fig. 2a).

Intracellular survivability of the isolates was also investigated at different time points (6, 10, 16, 24, 36 and 48 hours) in murine macrophage RAW264.7 cells. Initial reduction in the number of viable intracellular bacterial cells after phagocytosis was observed in all of the tested strains, which is in agreement with previous findings^11^(Fig. 2b). MRSA1679a demonstrated remarkable characteristics for intracellular survivability in murine macrophage RAW 264.7. At each tested time point, MRSA1679a survival rate was significantly higher than reference strain (P ≤ 0.004 at 6 h, P ≤ 0.019 at 10 h, P ≤ 0.048 at 16 h, P ≤ 0.035 at 24 h, P ≤ 0.039 at 36 h and P ≤ 0.011 at 48 h). In addition, MRSA1679a strain was not cleared from macrophages after 48 hours, unlike all strains.

### Murine model of *S. aureus* sepsis

In order to validate the *in vitro* results demonstrating the highly virulence characteristics of MRSA animal-isolates *in vitro*, we moved forward with an *in vivo* experiment with a murine model of *S. aureus* sepsis. The mouse lethality was significantly different for strain MRSA1679a. The LD_50_ experiment illustrated that MRSA1679a caused highest mortality rate and was the most virulent strain among the four tested strains with a mean LD_50_ of only 1.98 × 10^6^. There was no significant difference in the mean LD_50_ values for other strains when compared with the reference strain. MSSA (586 and 1161a) had mean LD_50_ values of 8.57 × 10^9^ and 2.49 × 10^9^, respectively. This result was not significant when compared with that of ATCC 29213 strain (2.17 × 10^8^). Also, MRSA478 isolate exhibited a non-significant LD_50_ value of 3.19 × 10^8^ when compared with the reference strain.

### RNA-seq Based Transcriptome Analysis

In order to gain additional insight into the different virulence regulators in MRSA isolates, we next moved to examine the gene expression profiles of the most virulent strain (MRSA1679a) and compared its transcriptome profile to the reference strain (ATCC 29213). RNA-seq was performed on MRSA1679a and the reference strain, and a total of 43,604,540 reads were obtained with a clean ratio of 98.6%. After mapping the reads, a total of 2,531 transcripts were identified in the MRSA1679a strain. When compared with the reference strain ATCC 29213, 399 genes were differentially expressed and out of those, 230 (58%) were down-regulated and 169 (42%) were up-regulated in the selected isolate (Supplementary Tables S2 and S3). Heat map of differential expression is presented in Supplementary Fig. S1.

Gene Ontology (GO) classification and Kyoto Encyclopedia of Genes and Genomes (KEGG) pathway analysis were performed as bioinformatics tool to explore the potential roles of the differentially expressed genes (DEGs) in resistance and virulence mechanism. Out of 399 DEGs, 355 (89%) were assigned GO terms and were further classified into three groups including biological process, molecular function and cellular component. Of those 355 genes, 218 (61%) were in the biological process group, 102 (29%) were in the molecular function group and 35 (10%) were in the cellular component group. Regarding the KEGG analysis, 94 DEGs were found to be involved in 72 different pathways.

**Figure 3:**
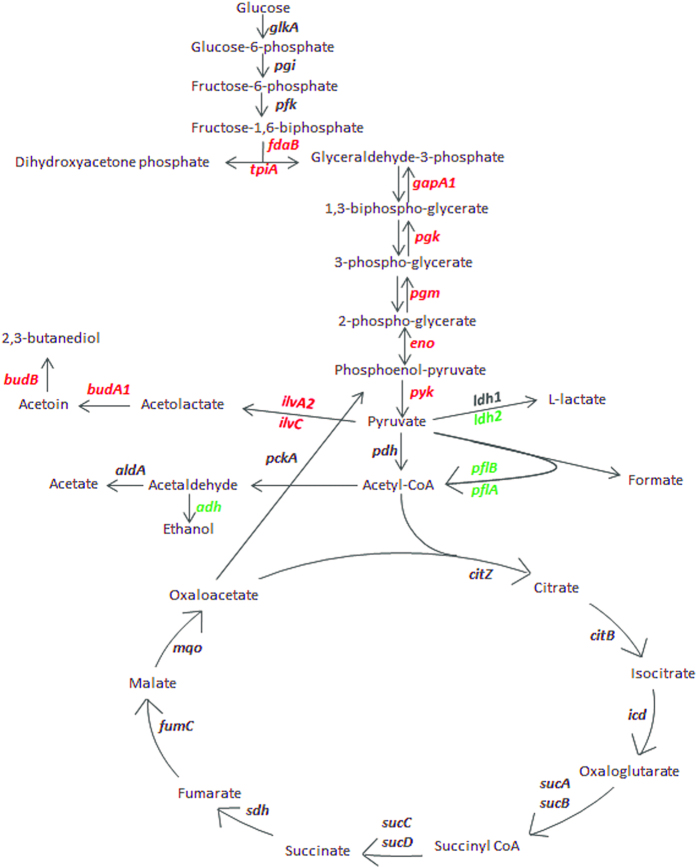
Differentially expressed genes associated with carbohydrate metabolism pathway in MRSA1679a. Up-regulated genes are marked as red, down-regulated as green while genes with no significant change are shown in black.

A good number of genes playing an important role in virulence, resistance and survival of *S. aureus* were significantly up-regulated in MRSA1679a (Table 2). These include genes encoding the intercellular adhesion proteins (*icaABCD*), *sdrE* adhesion protein, *LysM* protein, heme binding proteins (SAAV_0581 and SAAV_2631) and capsular proteins (*cap5ABCDEFGLMN*, SAAV_1044). Key regulators such as *sarA*, *kdpD*, *kdpE* and *thyA* were up-regulated in MRSA1679a. Many biofilm-related genes such as *eno*, *ureBCDEFG*, SAAV_1144, SAAV_1145, SAAV_1785 and SAAV_1946, and genes encoding the metal binding proteins such as manganese transport protein MntH, phosphotyrosine protein phosphatase and siderophore biosynthesis protein were also among DEGs. Genes involved in drug resistance mechanisms including *ermC*, and SAAV_1916 were upregulated. In addition, genes essential for bacterial survival such *cysK*, *trxA*, *prop*, SAAV_0149, SAAV_0833, SAAV_1344, SAAV_1732, SAAV_2243 and SAAV_2452 were up-regulated in differential gene expression. Furthermore, several genes involved in carbohydrate metabolism pathway were differentially expressed and most of those genes were up-regulated (Fig. 3).

In differential expression analysis with the reference strain, many important genes related to our area of interest were found to be down-regulated in the MRSA1679a strain (Table 3). These include many toxin producing genes (*efb*, *hlgB*, *hlgC*, *hlY*, *seg*, *sei*, *sem*, *sen*, *yent1*, *yent2*, SAAV_0195 and SAAV_1141), cell wall associated genes (*cap5H*, *cap5I*, *cap5J*, *sdrC*, *sdrD* and *isdA*) and two virulence regulating accessory gene regulators (*agrD* and *sarS*). One gene (SAAV_0105) involved in tetracycline resistance and six genes (*ctsR*, *dnaJ*, *dnaK*, *grpE*, *hrcA* and *clpB*) responsible for survival against environmental stress were down-regulated in the MRSA1679a test strain.

### Verification by RT-qPCR

Nine genes (*blaZ*, *ermC*, SAAV_1916, *sarA*, *sdrE*, *icaA*, *eno*, *thyA* and *ureF*) encoding proteins related to the resistance and virulence mechanism of *S. aureus* were selected for validation of RNA-seq results. Among the nine tested genes, only *blaZ* was not expressed in the reference strain. Other genes such as *eno*, *ermC*, *icaA*, SAAV_1916*, sarA*, *sdrE*, *thyA* and *ureF* had fold changes of 2.35, 591.79, 1.95, 4.12, 5.73, 9.38, 2.43 and 3.22, respectively when their expression levels were compared in both the test and reference strains. For these eight genes, trend of expression level by RT-qPCR was similar to that of RNA-seq (Supplementary Fig. S2) with a correlation coefficient of r = 0.959 which indicated a strong correlation between the two techniques (Supplementary Fig. S3).

## Discussion

Animal-derived isolates of MRSA are of both veterinary and medical interest and have been the main focus of extensive research in the last few years^12^. These livestock-associated MRSA are thought to have zoonotic potential^13^,^14^ and therefore, people in contact with livestock such as veterinarians, farmers and workers at slaughterhouses are at a great risk^15^. Farm animals can be a major ecological niche for the development of multidrug resistance in MRSA because of massive use of antibiotics leading to adaptation and evolution of the pathogen^16^. Despite the frequent reports of MRSA prevalence in animal-derived food products especially poultry retailed meat, there have been few studies regarding the genetic profiles of animal-associated MRSA strains. Due to the zoonotic potential of these MRSA isolates and the significant increase in their prevalence, improved efforts are required to study and understand the virulence potential of the multidrug-resistant strains through genomic and transcriptomic methods. In present study, we performed antimicrobial susceptibility as well as virulence study *in vitro* and *in vivo* of four livestock associated strains. Then, a highly virulent and resistant MRSA strain was selected to have insights into regulation of its virulence associated genes and resistance potential through transcriptome analysis.

Presently, more than 60% of all *S. aureus* isolates are methicillin-resistant and some of the MRSA strains have been reported to develop resistance against more than 20 different antimicrobials^17^. We tested livestock isolates for their antimicrobial susceptibility to 13 antimicrobial agents belonging to 8 different groups. Two of the isolates were found to be MRSA with increased resistance against almost all tested groups except aminoglycosides. In a recent report, poultry MRSA strains were resistant to 10 antibiotics of different groups^18^. In our study, MRSA1679a also showed resistance to 11 out of the 13 tested antimicrobial agents, displaying remarkable multidrug-resistant behavior.

Most of the multidrug-resistant MRSA strains have the capability of biofilm formation which is an important virulence determinant^19^ as well as a barrier against the treatment of infections^20^. Bacterial biofilm is a structured aggregation of bacterial cells surrounded by a self-produced matrix layer and adherent to the host surface^21^. We observed that *S. aureus* biofilms were time-dependent and both MRSA strains (478 and 1679a) had higher capability of biofilm formation as compared to the MSSA (586 and 1161a) strains or reference human-isolate. Moreover, MRSA1679a strain had the strongest biofilm formation ability which is in line with the concept that biofilms enhance the antibiotic resistance capacity of the bacteria^22^.

Macrophages, member of the innate immune system, are the first line of host defense mechanism against invading pathogens^23^. Adhesion to the host tissue is the first and foremost function of *S. aureus* surface proteins^24^. *S. aureus* have the capacity to invade and survive inside different kinds of host cells including macrophages^25^. This immune evasion strategy of the bacteria contributes to the development of chronic and recurrent infections^23^. In our *in vitro* study, we observed that MRSA strains had more adhesion and invasion rate as compared to those of MSSA strains. MRSA1679a could survive within macrophages for more than 48 hours while other three strains could not persist the same period. The survival rates of all tested strains were higher as compared to earlier reports^11^. Strong biofilm formation of MRSA1679a may have enhanced immune evasion mechanisms^24^.

*In vivo* studies are critical to perform in order to validate *in vitro* results obtained for virulence of these strains. Utilizing *S. aureus* sepsis model, we confirmed that strain MRSA1679a is highly virulent strain. LD_50_ dose was significantly lower for MRSA1679a depicting a high virulence potential of the poultry strain. The results of the MIC, biofilm formation, *in vitro* and *in vivo* assays clearly indicated that MRSA1679a was the most virulent and resistant than the other three strains. So, this strain was selected for gene expression analysis.

With the development of genome-wide transcriptional analysis, it has been suggested that a composite regulatory network governs the expression of numerous virulence determinants in *S. aureus*^26^. *S. aureus* has a range of virulence factors, such as surface proteins responsible for adhesion and invasion of the host cells, exoproteins responsible for immune evasion mechanism, and a number of pore-forming and hemolytic toxins^27^. A coordinated expression of these virulence factors is essential for successful infection^28^. In our transcriptome analysis of MRSA1679a isolate, genes related to several surface proteins, exoproteins and 22 of hemolytic and enterotoxins were expressed. Differential analysis of the strains revealed down-regulation of several toxins and up-regulation of many capsular and adhesion proteins in MRSA1679a. This could be due to down-regulation of *agrD* gene of agr regulatory system which positively controls the expression of many degradative exoenzymes and toxins, but appears to repress numerous colonization factors^29^ such as surface proteins^30^. It has been suggested that adhesion proteins can be more important than exotoxins for staphylococcal pathogenesis^31^.

**Figure 4:**
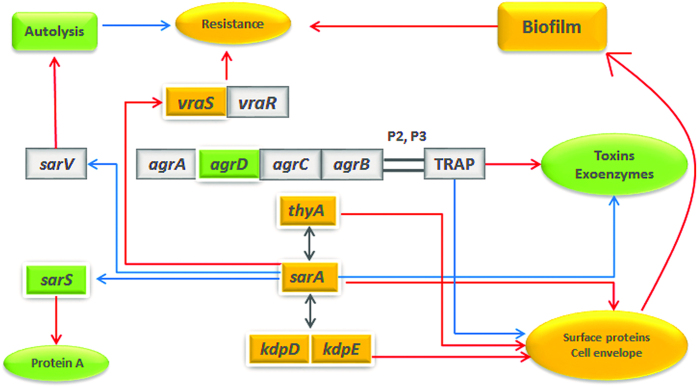
Schematic diagram of proposed virulence and resistance regulation in MRSA1679a. Up-regulation and down-regulation of genes and their products are shown in orange-yellow and green colors, respectively. Red arrows indicate positive regulation; blue arrows indicate negative regulation while double headed arrows point to correlation of two genes.

In addition to *sarA*, a two-component regulatory system in *S. aureus, KdpDE,* has also been linked to virulence factors as it increased the expression level of genes encoding for cell wall associated proteins and polysaccharides while repressed the transcription of toxin producing genes at the same time^8^. When compared with the reference strain, *sarA*, *KdpD* and *KdpE* were up-regulated and could be responsible for positive regulation of many adhesion and cell-wall protein associated genes. A recent study has identified that *thyA* is also involved in regulating the virulence determinants and have some positive linkages with *agr* and sarA^32^. Up-regulation of this gene was observed in the presence of increased expression level of *sarA*. Another accessory regulator *sarS* was down-regulated in MRSA1679a and might have been repressed by *sarA* as suggested by earlier studies^33^. In the light of available data and our gene expression profile, it is proposed that in addition to *agr* and *sarS*, *sarA* may also have some linkages with *KdpDE* and *thyA* to govern the virulence determinants as illustrated in Fig. 4.

Different iron acquisition mechanisms are also thought to play a key role in the infection process of *S. aureus*^34^,^35^. In our MRSA1679a strain, two genes encoding siderophore biosynthesis protein and ferrous iron transport protein B were up-regulated, suggesting their role in the virulence of the strain. Similarly, a gene encoding for manganese transport protein MntH was also significantly expressed in the bacteria and according to previous reports, MntH plays an important part for manganese acquisition during infection process of *S. aureus*^36^. Therefore, metals such as iron and manganese are the important elements for the virulence of *S. aureus* and may have played a key role during infection of mice in our LD_50_ experiment. Along with many other mobile genetic elements (MGEs), some highly expressed prophage related genes may also be responsible for the increased virulence of MRSA1679a. Phages can influence the expression of virulence factors and thus, play an important role in staphylococcal diversity^37^.

In our biofilm assay, MRSA1679a was identified as a strong biofilm producer and this was verified by up-regulation of many biofilm-associated genes. Those included adhesion proteins such as *icaABCD*, *eno*, *sdrE* and *lysM,* polysaccharides involving the cap5 operon, the *ure* operon (BCDEFG) encoding for urease enzymes and N-acetylmuramoyl-L-alanine amidase (*AM*). Our results have also shown up-regulation of genes related to the production of acetoin and 2,3-butanediol which are thought to be more important for biofilm formation under the direct control of *sarA* regulator^31^. An extracellular amyloid fibril, composed of small peptide molecules called phenol-soluble modulins (PSMs), has been found in biofilm matrix of all MRSA. PSM as a novel family of toxins contribute to biofilm development and spreading of biofilm associated MRSA infections^38^. Increased expression of two PSM genes also reflected the MRSA1679a biofilm forming capacity.

ATP-binding cassette (ABC) multidrug transporters are the primary class of multidrug transporters and responsible to pump multiple drugs out of the bacterial cell by utilizing the free energy of ATP hydrolysis^39^. In MRSA1679a, a gene encoding for multidrug ABC transporter ATP-binding protein was highly expressed and may have conferred resistance to a wide range of antimicrobial agents including those tested in our MIC experiment. Additionally, we also observed increased expression of other resistance related genes. Those include *ermC* as responsible for the resistance against macrolide-lincosamine-streptogramin B, *blaZ* that confers resistance against penicillins^40^ and *femA* which was reported to be essential for high level of resistance against methicillin in MRSA^41^. *blaZ* and *ermC* are plasmid encoded genes which show the role of MGEs in the development of antibiotic resistance.

The principal virulence regulator, *sarA*, is also responsible for the bacterial survival in the host and environment as it responds to changes in redox-potential and pH^42^ by controlling the expression of several genes such as *sodA*^43^, *budA* and *budB*. In MRSA1679a strain, expression of these stress related genes increased by 2, 10.6 and 6.9 fold, respectively. Production of acetoin and 2,3-butanediol is induced by *budA* and *budB* genes, and thought to be more crucial for acid tolerance instead of the acidic products of the pyruvate metabolism^44^. In addition to *budA* and *budB*, up-regulation of many other genes involved in carbohydrate metabolism (Fig. 3) could be important for stress tolerance by increasing the fermentation products. Several other important genes for bacterial survival had a positive differential expression and those include *trxA* and *cysK* genes responsible for managing oxidative stress^45^, *prop* gene important for the bacterial survival in host body^46^, *ImpB/MucB/SamB* encoding genes responsible for protection against UV rays^47^, *Ohr* family gene crucial for organic hydroperoxide resistance^48^, and *ureBCDEFG* genes for urease activity essential in acid shock mechanism^49^. In contrast, many important stress related genes were down-regulated and among those were the ATP-dependent *Clp* protease B, molecular chaperones *dnaJ* and *dnaK*, heat-shock protein *grpE*, transcriptional regulator *ctsR* and heat-inducible transcription repressor *hrcA*. Possible reason for higher expression of these genes in the control strain can be the presence of many toxins which might have led to stress on the bacterial cell^31^.

Concluding our findings, it can be speculated that MRSA1679a is a highly virulent as well as multidrug-resistant poultry associated strain and poses a serious threat to public health. It has a variety of virulence elements including a large number of toxins which can be dangerous in acute form of infection with decreased susceptibility to most of the commonly used antibiotics. More attentions are needed for the study of animal-associated MRSA strains to investigate the virulence potential of these emerging strains.

## Materials and Methods

### Bacterial Strains

Two MRSA (478 and 1679a isolated from pig and chicken, respectively) and two MSSA (586 and 1161a both isolated from pig) isolates were used in this study (Supplementary Table S1). *S. aureus* ATCC 29213 was used as a reference strain in all of the experiments. Routinely, the isolates were cultured for 24–48 hours on Mueller-Hinton (MH) Agar supplemented with 5% sheep blood (Ruite bio-technology limited company, Guangzhou, China) at 37 °C under microaerobic conditions (5% O_2_, 10% CO_2_, and 85% N_2_).

### Species Confirmation and Antimicrobial Susceptibility Test

Strains were cultured on selective Staphylococcus Chromogenic Medium (Qindao hope bio-technology limited company, China) and for species confirmation, PCR amplification of *nuc* gene was carried out^50^. Minimum inhibitory concentrations (MICs) for four isolates and the reference strain (ATCC 29213) were determined for oxacillin, ampicillin, methicillin, ceftiofur, tetracycline, ciprofloxacin, levofloxacin, sulfamethoxazole-trimethoprim, clindamycin, lincomycin, erythromycin, azithromycin and gentamicin by using agar dilution method as recommended by the Clinical and Laboratory Standards Institute (CLSI) M31-A3 guidelines.

### Biofilm Assay

Crystal violet staining was performed to measure biofilm formation by *S. aureus* isolates. Three independent experiments with three repeats were carried out for each strain. Describing briefly, 20 μL of bacterial log phase culture was added to 200 μL fresh MH broth in 96-well flat-bottom microtiter plates. The plates were incubated at 37 °C for 24, 48 and 72 hour time intervals under microaerobic conditions. After incubation, optical densities (ODs) of bacterial growths were measured at a wavelength of 630 nm and then each well was washed thrice with phosphate-buffered saline (PBS) to remove the planktonic cells. Subsequently, 200 μL of methanol was added to each well and plates were dried for 15 min at room temperature. The plates were again incubated at room temperature for 5 min after addition of 200 μL 10 g/L Hucker crystal violet solution. To remove unbound stain, wells were again washed with PBS and dried at 60 °C. Bound crystal violet was dissolved by treatment with 330 ml/L glacial acetic acid for 10 min and OD_570_ was measured for the stained bacteria and control wells. Biofilm formation index (BFI) was calculated and quantitative classification of biofilm formation was done as described earlier^51^.

### Adhesion, Invasion and Intracellular Survivability Assays

Murine macrophage RAW264.7 cells were cultured in Dulbecco’s Modified Eagle’s Medium (DMEM), supplemented with 10% fetal bovine serum (FBS), 2 mM L-glutamine, 10000 IU/mL penicillin and 10 mg/L streptomycin. The cells were grown routinely in tissue culture flasks at 37 °C in 5% CO_2_ humidified atmosphere. For all experimental assays, 24-well tissue culture plates were seeded with approximately 5.0 × 10^5^ cells/mL RAW264.7 cells and incubated for 24 hours prior to infection. Immediately before use, cell monolayers were washed twice with DMEM containing l% FBS and without antibiotics.

Each strain was cultured on MH blood agar plates at 37 °C microaerobically without antibiotic and harvested in DMEM containing 1% FBS (Hyclone, USA). Approximately, 5.0 × 10^6^ CFU/mL of the four selected isolates (478, 586, 1161a and 1679a) and the reference stain (ATCC29213) were inoculated into plate wells containing monolayer of cells. The infected macrophage monolayers were incubated as described before^52^. For the determination of total adherent and internalized bacteria, the cell monolayers were washed thrice with DMEM without antibiotic to flush the unbound extracellular bacteria. The cells were lysed with ice cold 0.2% Triton X-100 in sterile PBS (pH 7.2) to release the intracellular bacteria. The number of adherent and released intracellular bacteria was then counted and summarized after plating lysates on blood agar.

To determine the invading bacteria, cells were washed twice with DMEM and a medium containing 100 mg/ml of gentamicin was added for 1 hour to kill the extracellular bound bacteria. Then, the cells were washed thrice with DMEM and lysed with ice cold 0.2% Triton X-100 in sterile PBS (pH 7.2). Released intracellular bacteria were counted on blood agar plates after application of the lysate. The number of the internalized bacteria was subtracted from the total bacterial count to find the number of adherent bacteria. For the intracellular survivability test, the post-infection invasion period was extended to 6, 10, 16, 24, 36 and 48 hours for each bacterial strain. Cells were washed, lysed and serially diluted as described above. The gentamicin-specific MICs were determined for each strain using the agar dilution method as recommended by the CLSI M31-A3 guidelines.

### Murine model of *S. aureus* sepsis

The animal care and all experiments were approved and performed in accordance with the guidelines and regulations approved by the Hubei Science and Technology Agency Animal Care and Use Committee in China (SYXK 2013-0044). Female BALB/c mice were used for the LD_50_ study as described before^52^ and were purchased from the Center of Laboratory Animals of Hubei Province (Wuhan, China) and kept under specific pathogens free (SPF) conditions. Mice were divided into five groups (five bacterial isolates) and each group was further divided into six subgroups (six concentrations (10^6^–10^11^ cfu/ml) of each bacterial strain). Serial dilutions were prepared in sterile PBS and injected intraperitoneally. Infected mice were monitored for mortality for 7 days^11^. The 7 day survival ratios from two independent experiments were pooled for estimation of the median lethal dose (LD_50_) as described before^53^.

### RNA-seq Based Transcriptome Analysis

MRSA1679a was the most virulent and resistant *S. aureus* strain according to virulence and antibiotic susceptibility tests and, thus, was selected for transcriptome analysis. Four samples including two for each of MRSA1679a and the reference strain (ATCC 29213) were prepared and harvested at log phase. Total RNAs were extracted with TRIzol (Invitrogen Inc., California, USA) from the bacterial isolates according to the manufacturer’s instructions. The remaining DNA was removed by RNase-free DNase I (Ambion Inc., Texas, USA). RNA quality was tested by Agilent 2100 system with RIN (RNA integrity number) over 7. Ribosomal RNA was removed from the total RNA with Ribozero Kit followed by strand specific RNA-seq protocol on Illumina HiSeq2500 platform (paired-end sequencing; 100 bp fragments) at Shanghai Biochip Corporation. Briefly, first strand cDNA synthesis was performed by using SuperScriptII (Invitrogen, Carlsbad, CA) in the presence of random hexamer primers and the second strand cDNA was synthesized before end-repair and dA-tailing. DNA fragment ligation was carried out with TruSeq adapter and then amplified with TruSeq PCR primers for sequencing. Reads longer than 35 nt and  ≤2 N (ambiguous nucleotides) were retained. Moreover, paired reads which got mapped to sliva database (http://www.arb-silva.de/download/arb-files/) were removed.

Expression of each gene in different samples was transformed to CPG (counts per gene) by DESeq package using blind and fit-only parameter^54^. Mean CPG of gene expression were calculated for MRSA1679a and the reference strain from their respective repeats and compared to determine differentially expressed genes between the two strains. The transcripts with a P-value of ≤0.05 and a fold change of 2≥ were considered as differentially expressed. The data have been deposited in Gene Expression Omnibus (GEO) and are accessible through accession number GSE78764 (http://www.ncbi.nlm.nih.gov/geo/query/acc.cgi?acc=GSE78764). Gene Ontology (GO), being an international standardized system for a functional classification of genes, provides an updated terminology and comprehensively describes the properties of genes and their products in any organism^55^. DEGs were further analyzed using the three structured terminologies (ontologies) including biological process, molecular function and cellular component which reliably described the gene products. Similar to GO enrichment analysis, Kyoto Encyclopedia of Genes and Genomes (KEGG) database (http://www.genome.jp/kegg) was utilized to find out the linkage of the differential genes with various pathways^56^.

### Validation by RT-qPCR

For verification of RNA sequencing results, nine of the genes with increased expression in MRSA1679a were selected on the basis of their importance as resistance and virulence determinants. These included *blaZ* (β-lactamase), *ermC* (rRNA adenine N-6-methyltransferase), SAAV_1916 (multidrug ABC transporter ATP-binding protein), *sarA* (accessory regulator A), *sdrE* (sdrE protein), *icaA* (intercellular adhesion protein A), *ureF* (urease accessory protein), *thyA* (thymidylate synthase) and *eno* (phosphopyruvate hydratase). Thermonuclease precursor (*nuc*) gene was used as a housekeeping gene and RT-qPCR was performed as described earlier^57^. Primers used in RT-qPCR are given in Supplementary Table S4.

### Statistical Analysis

Statistical analyses were performed by using SPSS version 22.0 (IBM Corp., Armonk, NY, USA). LD_50_ values were calculated by probit analysis and two-tailed t-test was applied to estimate the mean ± standard deviation (MSD) and significance level among different strains in LD_50_, biofilm formation, adhesion, invasion and intracellular survivability assays. To compare the results of RNA-seq and RT-qPCR, a correlation coefficient (r) was determined by Pearson’s analysis. P-values of  ≤  0.05 were considered significant.

### Ethic Statement

The use of mice in this study was according to relevant guidelines and regulations of Animal Care Center, Hubei Science and Technology Agency in China (SYXK 2013-0044). Animal housing care and experimental protocol was according to the regulation of experimental animal usage in Hubei province, China.

## Additional Information

**How to cite this article**: Iqbal, Z. *et al*. Comparative virulence studies and transcriptome analysis of *Staphylococcus aureus* strains isolated from animals. *Sci. Rep.* **6**, 35442; doi: 10.1038/srep35442 (2016).

## Supplementary Material

Supplementary Information

## Figures and Tables

**Table 1:** MICs of *S. aureus* isolates against different antibiotics.

Strains	MICs (mg/L) against different antimicrobial agents												
	OXA	AMP	MET	CEF	TET	CIP	LEV	SXT	CLI	LIN	ERY	AZM	GEN
478	32	1	64	4	64	4	2	32/608	256	>512	512	512	4
586	0.25	8	0.25	4	64	4	2	16/304	512	>512	512	512	8
1611a	0.25	1	2	32	32	0.25	0.25	128/2432	0.125	0.5	0.5	0.5	0.25
1679a	8	4	32	128	0.5	32	16	128/2432	>512	>512	512	512	0.25
ATCC29213	0.125	0.125	0.25	4	0.5	0.25	0.125	<2/38	<0.125	0.5	<0.5	<0.5	0.5

OXA: oxacillin; AMP: ampicillin; MET: methicillin; CEF: ceftiofur; TET: tetracycline; CIP: ciprofloxacin; LEV: levofloxacin; SXT: sulfamethoxazole-trimethoprim; CLI: clindamycin; LIN: lincomycin; ERY: erythromycin; AZM: azithromycin; GEN: gentamicin.

**Table 2:** Important up-regulated genes in MRSA1679a during differential expression analysis.

Gene/ORF	Gene product	P-value	Fold change
Virulence			
*icaA*	intercellular adhesion protein A	4.32E-05	9.12
*icaB*	intercellular adhesion protein B	0.049359	3.12
*icaC*	intercellular adhesion protein C	0.042855	3.44
*icaD*	intercellular adhesion protein D	0.019102	5.35
*sdrE*	sdrE protein	0.000141	5.59
*eno*	phosphopyruvate hydratase	0.014628	2.85
*ureB*	urease subunit beta	0.002621	4.16
*ureC*	urease subunit alpha	0.000846	4.52
*ureD*	urease accessory protein UreD	0.00663	3.34
*ureE*	urease accessory protein UreE	0.002252	4.13
*ureF*	urease accessory protein UreF	0.002263	4.05
*ureG*	urease accessory protein UreG	0.001419	4.26
*cap5A*	capsular polysaccharide biosynthesis proteinCap5A	2.05E-06	8.65
*cap5B*	capsular polysaccharide biosynthesis proteinCap5B	8.29E-06	7.46
*cap5C*	capsular polysaccharide biosynthesis proteinCap5C	4.07E-05	6.23
*cap5D*	capsular polysaccharide biosynthesis proteinCap5D	6.79E-05	5.83
*cap5E*	capsular polysaccharide biosynthesis proteinCap5E	0.00033	4.97
*cap5F*	capsular polysaccharide synthesis enzyme Cap5F	0.000144	5.52
*cap5G*	UDP-N-acetylglucosamine 2-epimerase Cap5G	2.73E-05	6.75
*cap5L*	capsular polysaccharide biosynthesis proteinCap5L	0.000208	5.41
*cap5M*	capsular polysaccharide biosynthesis galactosyltransferase Cap5M	0.001864	4.11
*cap5N*	capsular polysaccharide biosynthesis proteinCap5N	0.002776	3.86
SAAV_0581	heme binding proteins	0.036714	2.44
SAAV_0718	LysM domain-containing protein	2.50E-05	7.45
SAAV_1044	cell-wall binding lipoprotein	0.000758	4.73
SAAV_1071	manganese transport protein MntH	0.000538	4.71
SAAV_1144	anti-protein (phenol soluble modulin)	0.044331	2.30
SAAV_1145	anti-protein (phenol soluble modulin)	0.015271	2.76
SAAV_1785	N-acetylmuramoyl-L-alanine amidase	0.037926	2.67
SAAV_1946	phosphotyrosine protein phosphatase	0.036493	2.48
SAAV_2242	siderophore biosynthesis protein	0.029779	2.48
SAAV_2616	ferrous iron transport protein B	0.007373	3.59
SAAV_2631	heme binding proteins	0.005153	3.45
Regulator			
*kdpD*	sensor histidine kinase KdpD	0.017446	2.91
*kdpE*	DNA-binding response regulator KdpE	0.049855	2.49
*sarA*	accessory regulator A	0.018333	2.71
*thyA*	thymidylate synthase	0.046559	2.34
Resistance			
*ermC*	rRNA adenine N-6-methyltransferase	0.000213	1500
SAAV_1916	multidrug ABC transporter, permease	0.013189	3.16
Stress			
*cysK*	cysteine synthase	0.013471	3.00
*trxA*	thioredoxin	0.013116	2.87
*proP*	osmoprotectant proline transporter	0.00601	3.44
*budA1*	alpha-acetolactate decarboxylase	4.74E-07	10.56
*budB*	acetolactate synthase	2.34E-05	6.90
SAAV_0149	membrane protein YagU	0.004216	3.52
SAAV_1344	ImpB/MucB/SamB family protein	0.000756	4.81
SAAV_1732	OsmC/Ohr family protein	0.010857	3.14
Prophage			
SAAV_0833	Siphovirus Gp157	2.28E-24	943.00
SAAV_0834	phage single-strand DNA binding protein	3.48E-06	9.28
SAAV_0838	phage replication protein	5.81E-05	6.94
SAAV_0844	conserved hypothetical phage protein	0.001692	4.59

**Table 3:** Important down-regulated genes in MRSA1679a during differential expression analysis.

Gene/ORF	Gene product	P-value	Fold change
Virulence			
*efb*	fibrinogen-binding protein	0.00565	0.23
*hlgB*	gamma hemolysin, component B	0.016243	0.31
*hlgC*	gamma hemolysin, component C	0.026725	0.33
*hlY*	alpha-hemolysin precursor	1.13E-05	0.12
*isaB*	immunodominant antigen B	4.42E-05	0.15
*isdA*	LPXTG cell wall surface anchor protein	0.013414	0.30
*plc*	1-phosphatidylinositol phosphodiesterase	0.033811	0.29
*sdrC*	sdrC protein	0.00747	0.30
*sdrD*	sdrD protein	0.013505	0.32
*seg*	enterotoxin G	2.61E-09	0.03
*sei*	enterotoxin I	7.25E-08	0.03
*sem*	enterotoxin M	1.39E-05	0.06
*sen*	enterotoxin N	1.28E-09	0.02
*yent1*	enterotoxin Yent1	5.71E-07	0.03
*yent2*	enterotoxin Yent2	1.16E-05	0.03
*cap5H*	capsular polysaccharide biosynthesis proteinCap5H	1.23E-07	0.04
*cap5I*	capsular polysaccharide biosynthesis proteinCap5I	3.18E-10	0.02
*cap5J*	capsular polysaccharide biosynthesis proteinCap5J	6.68E-09	0.04
SAAV_0367	superantigen-like protein	0.035917	0.30
SAAV_0369	superantigen-like protein	0.038693	0.25
SAAV_0376	superantigen-like protein	0.004694	0.10
SAAV_0843	PVL ORF-50 family protein	0.000431	0.18
SAAV_1134	superantigen-like protein	2.99E-06	0.09
SAAV_1135	superantigen-like protein	1.21E-06	0.08
SAAV_1136	superantigen-like protein	4.69E-08	0.06
SAAV_1141	exfoliative toxin, putative	0.013705	0.33
SAAV_2484	IgG-binding protein SBI	1.76E-05	0.13
SAAV_2562	LPXTG-motif protein	8.15E-12	0.03
SAAV_2661	LPXTG-motif protein	0.00478	0.10
Regulator			
*agrD*	accessory gene regulator protein D	1.55E-15	0.02
*sarS*	accessory regulator S	0.027637	0.37
Resistance			
SAAV_0105	tetracycline resistance protein, putative	0.001764	0.25
Stress			
*ctsR*	transcriptional regulator CtsR	0.005163	0.28
*dnaJ*	chaperone protein DnaJ	0.024337	0.37
*dnaK*	molecular chaperone DnaK	0.008807	0.31
*grpE*	heat shock protein GrpE	0.000402	0.20
*hrcA*	heat-inducible transcription repressor HrcA	7.93E-05	0.17
*clpB*	ATP-dependent Clp protease, ATP-binding subunitClpB	0.000324	0.20
Prophage			
SAAV_0827	phage anti repressor	0.031911	0.45
SAAV_0867	phage tail tape measure protein	4.09E-07	0.10
SAAV_0869	phage minor structural protein	1.94E-07	0.09
SAAV_0874	phage amidase	3.16E-12	0.02
