# Supplementary material for: Comparative virulence studies and transcriptome analysis of Staphylococcus aureus strains isolated from animals
Source: Sci Rep. 2016 Oct 14;6:35442. doi: 10.1038/srep35442 (PMC5064352; doi:10.1038/srep35442)

Comparative virulence studies and transcriptome analysis of *Staphylococcus aureus* strains isolated from animals

Zahid Iqbal, Mohamed N. Seleem, Hafiz Iftikhar Hussain, Lingli Huang, Haihong Hao, Zonghui Yuan

Supplementary tables and figures

Supplementray tables

Supplementary table S1. Bacterial isolates used in this study.

Strain	Source	Place	Year	Resistance status
S. aureus 478	Pig	Hunan, China	2012	MRSA
S. aureus 586	Pig	Hunan, China	2012	MSSA
S. aureus 1161a	Pig	Hubei, China	2012	MSSA
S. aureus 1679a	Chicken	Henan, China	2012	MRSA

Supplementary table S2. Down-regulated genes in MRSA1679a

Gene	Fold Change	P-value	Gene product
adhP	0.295855	0.007658	alcohol dehydrogenase
agrD	0.016466	1.55E-15	accessory gene regulator protein D
ald1	0.027148	5.35E-13	alanine dehydrogenase
arcA	0.087213	2.75E-07	arginine deiminase
arcB1	0.034469	1.06E-11	ornithine carbamoyltransferase
arcB2	0.057852	5.30E-09	ornithine carbamoyltransferase
arcC1	0.033298	7.48E-12	carbamate kinase
arcC2	0.057306	4.86E-09	carbamate kinase
arcD	0.059322	7.69E-09	arginine/ornithine antiporter
argH	0.276573	0.003848	argininosuccinate lyase
betA	0.395523	0.04034	choline dehydrogenase
cap5H	0.041025	1.23E-07	capsular polysaccharide biosynthesis proteinCap5H
cap5I	0.02452	3.18E-10	capsular polysaccharide biosynthesis proteinCap5I
cap5J	0.035582	6.68E-09	capsular polysaccharide biosynthesis proteinCap5J
clpB	0.196235	0.000324	ATP-dependent Clp protease, ATP-binding subunitClpB
ctsR	0.285206	0.005163	transcriptional regulator CtsR

deoC1	0.383581	0.035632	deoxyribose-phosphate aldolase
deoD1	0.311143	0.009331	purine nucleoside phosphorylase
dnaJ	0.371089	0.024337	chaperone protein DnaJ
dnaK	0.313789	0.008807	molecular chaperone DnaK
drp35	0.326386	0.012592	drP35 protein
efb	0.226677	0.00565	fibrinogen-binding protein
glpT	0.332604	0.014057	glycerol-3-phosphate transporter
gltS	0.291444	0.006504	sodium:glutamate symporter
grpE	0.205332	0.000402	heat shock protein GrpE
hlgB	0.312011	0.016243	gamma hemolysin, component B
hlgC	0.33147	0.026725	gamma hemolysin, component C
hlY	0.122051	1.13E-05	alpha-hemolysin precursor
hrcA	0.172038	7.93E-05	heat-inducible transcription repressor HrcA
hsdS	0.018881	2.50E-14	type I restriction-modification enzyme, Ssubunit
hutH	0.276825	0.004904	histidine ammonia-lyase
hutI	0.118475	5.83E-06	imidazolonepropionase
hutU	0.165849	0.000117	urocanate hydratase
ilvA1	0.024217	1.57E-13	threonine dehydratase
isaB	0.151656	4.42E-05	immunodominant antigen B
isdA	0.305511	0.013414	LPXTG cell wall surface anchor protein
ldh2	0.26612	0.003913	L-lactate dehydrogenase
modA	0.18892	0.00025	molybdenum ABC transporter, periplasmicmolybdate-binding protein
modB	0.224719	0.001086	molybdenum ABC transporter permease
modC	0.260512	0.003168	molybdenum ABC transporter, ATP-binding proteinModC
murQ	0.329364	0.017011	N-acetylmuramic acid-6-phosphate etherase
nanA	0.265967	0.00435	N-acetylneuraminate lyase
pdp	0.317609	0.012586	pyrimidine-nucleoside phosphorylase
pflA	0.297446	0.006769	pyruvate formate-lyase-activating enzyme
pflB	0.376365	0.027491	formate acetyltransferase
plc	0.286302	0.033811	1-phosphatidylinositol phosphodiesterase
prmA	0.38727	0.028958	ribosomal protein L11 methyltransferase
SAAV_0027	0.404154	0.042236	5'-nucleotidase family protein
SAAV_0030	0.045359	2.73E-10	hypothetical protein
SAAV_0032	0.271827	0.006359	hypothetical protein
SAAV_0034	0.107934	5.37E-06	hypothetical protein
SAAV_0035	0.202721	0.000555	Zn-dependent hydrolase
SAAV_0039	0.258609	0.025533	hypothetical protein
SAAV_0041	0.031711	6.46E-12	hypothetical protein
SAAV_0042	0.329597	0.015552	hypothetical protein
SAAV_0044	0.369292	0.038424	hypothetical protein

SAAV_0105	0.247111	0.001764	tetracycline resistance protein, putative
SAAV_0111	0.317422	0.033886	phosphonate ABC transporter substrate-bindingprotein
SAAV_0161	0.282907	0.006387	PTS system, IIBC components
SAAV_0162	0.264048	0.004186	RpiR family phosphosugar-binding transcriptionalregulator
SAAV_0164	0.027656	0.047307	hypothetical protein
SAAV_0165	0.051993	0.008542	hypothetical protein
SAAV_0166	0.039418	1.17E-05	ABC transporter permease
SAAV_0167	0.014896	1.24E-10	hypothetical protein
SAAV_0168	0.047098	0.004769	hypothetical protein
SAAV_0169	0.035914	0.037787	hypothetical protein
SAAV_0170	0.092045	0.021579	putative ABC transporter, ATP-binding protein
SAAV_0185	0.407464	0.049022	hypothetical protein
SAAV_0195	0.347178	0.03866	coagulase family-protein
SAAV_0208	0.182029	0.00022	PTS system, IIBC components
SAAV_0248	0.317221	0.008138	hypothetical protein
SAAV_0264	0.032366	1.18E-08	hypothetical protein
SAAV_0274	0.375387	0.036709	5'-nucleotidase
SAAV_0278	0.110392	8.25E-06	carbohydrate kinase
SAAV_0279	0.06257	3.12E-08	hypothetical protein
SAAV_0280	0.073972	1.41E-07	nucleoside permease NupC, putative
SAAV_0281	0.141782	3.77E-05	sodium:solute symporter family protein
SAAV_0286	0.332743	0.034789	hypothetical protein
SAAV_0287	0.235962	0.001806	lipase precursor, interruption-N
SAAV_0336	0.063796	2.07E-05	hypothetical protein
SAAV_0361	0.117132	0.001376	IS3 family transposase
SAAV_0362	0.219114	0.033246	hypothetical protein
SAAV_0363	0.323656	0.023747	hypothetical protein
SAAV_0367	0.300883	0.035917	superantigen-like protein
SAAV_0369	0.24947	0.038693	superantigen-like protein
SAAV_0376	0.103719	0.004694	superantigen-like protein
SAAV_0383	0.029088	4.28E-05	putative lipoprotein
SAAV_0484	0.31855	0.010469	hypothetical protein
SAAV_0485	0.31909	0.01063	ATP:guanido phosphotransferase
SAAV_0540	0.10481	1.79E-06	hypothetical protein
SAAV_0541	0.12436	8.54E-06	hypothetical protein
SAAV_0560	0.380726	0.031502	hypothetical protein
SAAV_0561	0.03253	8.55E-10	hypothetical protein
SAAV_0594	0.287932	0.006091	ABC transporter substrate-binding protein
SAAV_0595	0.255428	0.002664	ABC transporter permease
SAAV_0596	0.258425	0.002473	ABC transporter ATP-binding protein

SAAV_0653	0.340803	0.01512	anion transporter family protein
SAAV_0698	0.359736	0.023937	transferrin receptor
SAAV_0758	0.150971	0.000612	putative secreted von Willebrand factor-bindingprotein
SAAV_0772	0.378328	0.038922	LysE/YggA family protein
SAAV_0818	0.16237	0.004257	integrase
SAAV_0821	0	2.95E-05	hypothetical protein
SAAV_0822	0	3.14E-35	putative phage repressor
SAAV_0824	0	4.53E-18	hypothetical protein
SAAV_0825	0	2.53E-09	hypothetical protein
SAAV_0826	0.123447	6.44E-05	hypothetical protein
SAAV_0827	0.445203	0.031911	phage anti repressor
SAAV_0829	0.042809	0.004448	hypothetical protein
SAAV_0831	0.284076	0.005109	hypothetical protein
SAAV_0836	0.402595	0.044773	hypothetical protein
SAAV_0837	0.075786	3.94E-08	hypothetical protein
SAAV_0841	0.126063	0.000671	putative resolvase
SAAV_0843	0.181263	0.000431	PVL ORF-50 family protein
SAAV_0845	0.031285	5.70E-11	putative dUTP pyrophosphatase
SAAV_0847	0.405722	0.045656	hypothetical protein
SAAV_0848	0.214326	0.000386	hypothetical protein
SAAV_0849	0.271406	0.035958	hypothetical protein
SAAV_0850	0.274822	0.00395	hypothetical protein
SAAV_0867	0.099808	4.09E-07	phage tail tape measure protein
SAAV_0868	0.087947	8.97E-08	hypothetical protein
SAAV_0869	0.094747	1.94E-07	phage minor structural protein
SAAV_0870	0.105092	7.74E-06	hypothetical protein
SAAV_0871	0.080276	5.78E-08	hypothetical protein
SAAV_0872	0.123598	2.62E-06	hypothetical protein
SAAV_0873	0.006875	3.99E-06	holin, SPP1 family
SAAV_0874	0.019281	3.16E-12	phage amidase
SAAV_0920	0.366514	0.021725	glycerophosphoryl diester phosphodiesteraseGlpQ, putative
SAAV_0990	0.352026	0.023094	ComK family protein
SAAV_0991	0.330943	0.015218	hypothetical protein
SAAV_1122	0.184891	0.000435	fibrinogen binding-related protein
SAAV_1127	0.249183	0.017225	fibrinogen-binding protein precursor-relatedprotein
SAAV_1129	0.176966	0.000254	hypothetical protein
SAAV_1130	0.1381	0.006186	IS1181 transposase
SAAV_1134	0.094008	2.99E-06	superantigen-like protein
SAAV_1135	0.081202	1.21E-06	superantigen-like protein
SAAV_1136	0.063375	4.69E-08	superantigen-like protein

SAAV_1139	0.075414	6.89E-08	hypothetical protein
SAAV_1141	0.327463	0.013705	exfoliative toxin, putative
SAAV_1293	0.065244	0.003387	hypothetical protein
SAAV_1306	0.345214	0.022822	aspartate kinase
SAAV_1371	0.258526	0.009027	phosphate ABC transporter substrate-bindingprotein
SAAV_1420	0.027146	6.84E-13	putative protein
SAAV_1421	0.0237	1.36E-13	amino acid permease
SAAV_1479	0.194069	0.000337	hypothetical protein
SAAV_1564	0.075184	3.77E-08	hypothetical protein
SAAV_1565	0.213739	0.000651	hypothetical protein
SAAV_1566	0.304278	0.007337	hypothetical protein
SAAV_1643	0.323384	0.015899	hypothetical protein
SAAV_1644	0.370128	0.021593	hypothetical protein
SAAV_1807	0.305182	0.009011	hypothetical protein
SAAV_1808	0.24534	0.00147	hypothetical protein
SAAV_1809	0.22012	0.000665	hypothetical protein
SAAV_1813	0.208936	0.015025	transposase
SAAV_1814	0.030657	3.41E-09	transposase
SAAV_1818	0.141184	0.037855	serine protease SplD, putative
SAAV_1830	0.081665	1.86E-06	hypothetical protein
SAAV_2009	0	9.42E-23	caax amino protease family protein; putativemembrane protein
SAAV_2011	0	6.87E-27	holin
SAAV_2033	0	0.000101	phage terminase small subunit
SAAV_2034	0.064074	5.42E-07	HNH endonuclease family protein
SAAV_2035	0.313249	0.005366	phage transcriptional regulator, RinA familyprotein
SAAV_2040	0.275642	0.049601	hypothetical protein
SAAV_2044	0	0.003616	hypothetical protein
SAAV_2053	0	0.012731	hypothetical protein
SAAV_2056	0.357978	0.01031	hypothetical protein
SAAV_2057	0	3.02E-10	hypothetical protein
SAAV_2063	0	9.84E-38	hypothetical protein
SAAV_2064	0	1.94E-60	hypothetical protein
SAAV_2069	0.001436	1.27E-24	hypothetical protein
SAAV_2165	0.420572	0.047804	hypothetical protein
SAAV_2204	0.023996	4.21E-05	putative transposase
SAAV_2264	0.098383	2.31E-06	M23/M37 peptidase domain-containing protein
SAAV_2265	0.262785	0.004173	hypothetical protein
SAAV_2266	0.325971	0.011808	surface protein, putative
SAAV_2269	0.029384	5.85E-13	hypothetical protein
SAAV_2270	0.019574	6.56E-15	hypothetical protein

SAAV_2271	0.034412	5.62E-07	hypothetical protein
SAAV_2272	0.021876	0.000902	putative ATP-binding protein
SAAV_2273	0.015741	0.045282	putative ATP-binding protein
SAAV_2275	0.057848	0.001676	putative transposase
SAAV_2405	0.369282	0.023725	hypothetical protein
SAAV_2431	0.335703	0.012167	L-lactate permease
SAAV_2440	0.038759	1.89E-11	hypothetical protein
SAAV_2443	0.24246	0.002323	PTS system, sucrose-specific IIBC components,putative
SAAV_2447	0.242861	0.011767	hypothetical protein
SAAV_2481	0.336394	0.01863	hypothetical protein
SAAV_2484	0.131242	1.76E-05	IgG-binding protein SBI
SAAV_2523	0.17082	0.000268	addiction module antitoxin
SAAV_2524	0.285284	0.007886	addiction module antitoxin
SAAV_2532	0.282193	0.00838	peptide ABC transporter permease
SAAV_2533	0.323904	0.013996	peptide ABC transporter substrate-bindingprotein
SAAV_2534	0.210054	0.01198	hypothetical protein
SAAV_2536	0.181017	0.010984	hypothetical protein
SAAV_2537	0.096675	1.24E-06	hypothetical protein
SAAV_2543	0.260164	0.007305	hypothetical protein
SAAV_2548	0.032844	2.69E-10	hypothetical protein
SAAV_2549	0.031733	8.23E-11	hypothetical protein
SAAV_2550	0.030415	1.88E-12	hypothetical protein
SAAV_2551	0.025792	3.09E-13	putative lipoprotein
SAAV_2556	0.249497	0.003696	MutT/nudix family protein
SAAV_2561	0.043138	1.42E-07	hypothetical protein
SAAV_2562	0.03064	8.15E-12	LPXTG-motif protein
SAAV_2577	0.368873	0.026635	transporter, putative
SAAV_2579	0.057338	1.51E-08	ABC transporter ATP-binding protein
SAAV_2580	0.159745	0.00016	putative membrane spanning protein
SAAV_2618	0.227479	0.001392	membrane protein
SAAV_2659	0.031211	5.15E-08	TetR family transcriptional regulator
SAAV_2661	0.097221	0.00478	lpxtg-motif protein
SAAV_2700	0.051314	1.57E-09	transcriptional regulator, Crp/Fnr family
SAAV_2707	0.158309	0.000408	transposase
SAAV_2709	0.121253	1.14E-05	hypothetical protein
SAAV_2710	0.374321	0.031002	BglG family transcriptional antiterminator
SAAV_2717	0.293833	0.005782	hypothetical protein
SAAV_2718	0.30577	0.007955	glycosyl transferase, group 1 family protein
SAAV_2719	0.253546	0.002204	preprotein translocase subunit SecA
SAAV_2727	0.373403	0.032787	hypothetical protein
SAAV_2778	0.22433	0.003551	

sarS	0.375394	0.027637	accessory regulator S
scdA	0.367186	0.02344	cell wall biosynthesis protein ScdA
sdrC	0.303753	0.00747	sdrC protein
sdrD	0.324498	0.013505	sdrD protein
seg	0.031756	2.61E-09	enterotoxin G
sei	0.035022	7.25E-08	enterotoxin I
sem	0.055566	1.39E-05	enterotoxin M
sen	0.018571	1.28E-09	enterotoxin N
serS	0.35516	0.020484	seryl-tRNA synthetase
sirA	0.345075	0.030056	iron compound ABC transporter, ironcompound-binding protein SirA
thrS	0.159583	8.93E-05	threonyl-tRNA synthetase
yent1	0.031677	5.71E-07	enterotoxin Yent1
yent2	0.033357	1.16E-05	enterotoxin Yent2

Supplementary table S3. Up-regulated genes in MRSA1679a

**Gene**	**Fold Change**	**P-value**	**Gene product**
bglA	4.6433	0.000792	6-phospho-beta-glucosidase
budA1	10.57335	4.74E-07	alpha-acetolactate decarboxylase
budB	6.908409	2.34E-05	acetolactate synthase
cap5A	8.658934	2.05E-06	capsular polysaccharide biosynthesis proteinCap5A
cap5B	7.46925	8.29E-06	capsular polysaccharide biosynthesis proteinCap5B
cap5C	6.233896	4.07E-05	capsular polysaccharide biosynthesis proteinCap5C
cap5D	5.829886	6.79E-05	capsular polysaccharide biosynthesis proteinCap5D
cap5E	4.968925	0.00033	capsular polysaccharide biosynthesis proteinCap5E
cap5F	5.52093	0.000144	capsular polysaccharide synthesis enzyme Cap5F
cap5G	6.738707	2.73E-05	UDP-N-acetylglucosamine 2-epimerase Cap5G
cap5L	5.417289	0.000208	capsular polysaccharide biosynthesis proteinCap5L
cap5M	4.106767	0.001864	capsular polysaccharide biosynthesisgalactosyltransferase Cap5M
cap5N	3.853611	0.002776	capsular polysaccharide biosynthesis proteinCap5N
cysK	2.900036	0.013471	cysteine synthase
eno	2.855882	0.014628	phosphopyruvate hydratase
ermC	1500	0.000213	rRNA adenine N-6-methyltransferase
gapA1	5.157691	0.000186	glyceraldehyde 3-phosphate dehydrogenase
glmS	3.35358	0.005971	glucosamine--fructose-6-phosphateaminotransferase
gntR	3.672139	0.004085	gluconate operon transcriptional repressor
hisC	2.427928	0.039405	histidinol-phosphate aminotransferase
icaA	9.12252	4.32E-05	Intercellular adhesion protein A

icaB	3.193401	0.049359	Intercellular adhesion protein B
icaC	3.443603	0.042855	Intercellular adhesion protein C
icaD	5.35025	0.019102	Intercellular adhesion protein D
ilvA2	5.260496	0.00037	threonine dehydratase
ilvC	2.855733	0.019461	ketol-acid reductoisomerase
kdpD	2.910431	0.017446	sensor histidine kinase KdpD
kdpE	2.491013	0.049855	DNA-binding response regulator KdpE
leuA	3.237637	0.009171	2-isopropylmalate synthase
leuB	5.228952	0.000355	3-isopropylmalate dehydrogenase
leuC	6.087064	0.000107	isopropylmalate isomerase large subunit
leuD	7.472121	6.09E-05	isopropylmalate isomerase small subunit
mtlD	2.373175	0.03519	mannitol-1-phosphate 5-dehydrogenase
mutY	3.049058	0.0189	A/G-specific adenine glycosylase
nixA	2.434648	0.041715	high-affinity nickel-transport protein
pgk	5.235937	0.000173	phosphoglycerate kinase
pgm	5.143043	0.00021	phosphoglyceromutase
proP	3.442846	0.00601	osmoprotectant proline transporter
rbsK	3.06864	0.011147	ribokinase
recX	3.104663	0.014772	recombination regulator RecX
SAAV_0046	1500	3.01E-05	putative lipoprotein, truncated
SAAV_0065	1500	0.017407	transposon-related protein
SAAV_0066	1500	2.34E-11	putative lipoprotein
SAAV_0067	1500	9.35E-07	Putative cell-wall binding lipoprotein
SAAV_0068	5.292306	0.001345	tandem lipoprotein
SAAV_0093	2.416214	0.045921	hypothetical protein
SAAV_0149	3.522127	0.004216	Uncharacterized membrane protein YagU
SAAV_0200	3.490097	0.005275	acetyl-CoA/acetoacetyl-CoA transferase
SAAV_0209	4.213409	0.001672	inosine-uridine preferring nucleoside hydrolase
SAAV_0210	8.093464	6.95E-06	BglG family transcriptional antiterminator
SAAV_0211	4.171489	0.002858	PTS system, sugar-specific IIA component,putative
SAAV_0212	5.038398	0.001263	PTS system, sorbitol-specific IIB component
SAAV_0214	4.261155	0.001551	PTS system, sorbitol-specific IIC component
SAAV_0215	3.579371	0.005046	sorbitol dehydrogenase
SAAV_0237	2.621022	0.033722	D-ribose pyranase
SAAV_0238	2.849004	0.019349	hypothetical protein
SAAV_0309	5.808593	0.001008	hypothetical protein
SAAV_0314	2.841707	0.023067	MttB family protein
SAAV_0316	2.661556	0.036412	hypothetical protein
SAAV_0341	2.522387	0.027711	hypothetical protein
SAAV_0342	2.443083	0.032354	hypothetical protein
SAAV_0378	5.740279	0.000188	hypothetical protein

SAAV_0380	4.227868	0.010163	putative lipoprotein
SAAV_0382	3.441695	0.023225	putative lipoprotein
SAAV_0417	6.765882	2.90E-05	PTS system, IIBC components
SAAV_0418	6.545452	3.71E-05	alpha-amylase family protein
SAAV_0419	4.681435	0.000641	GntR family transcriptional regulator
SAAV_0535	3.16511	0.021317	putative hydrolase, haloacid dehalogenase-likeprotein
SAAV_0579	2.38858	0.042761	alpha/beta fold family hydrolase
SAAV_0581	2.441008	0.036714	hypothetical protein
SAAV_0619	2.326904	0.047396	acetyltransferase
SAAV_0637	2.794771	0.029318	hypothetical protein
SAAV_0641	2.509893	0.034654	hypothetical protein
SAAV_0665	2.922421	0.013117	aldo/keto reductase family oxidoreductase
SAAV_0666	2.584257	0.026489	glycosyl transferase, group 2 family protein
SAAV_0718	7.459784	2.50E-05	LysM domain-containing protein
SAAV_0755	2.811154	0.03847	hypothetical protein
SAAV_0832	3.518156	0.011428	hypothetical protein
SAAV_0833	943.3276	2.28E-24	Siphovirus Gp157
SAAV_0834	9.28028	3.48E-06	putative phage single-strand DNA bindingprotein
SAAV_0838	6.940627	5.81E-05	putative phage replication protein
SAAV_0839	6.854713	6.25E-05	DnaC
SAAV_0840	5.498323	0.001527	hypothetical protein
SAAV_0842	13.15963	4.04E-06	hypothetical protein
SAAV_0844	4.587379	0.001692	conserved hypothetical phage prtotein
SAAV_0846	43.51237	1.85E-06	hypothetical protein
SAAV_0876	573.3209	1.19E-15	hypothetical protein
SAAV_0877	1414.857	6.87E-18	hypothetical protein
SAAV_0878	426.2194	3.91E-20	hypothetical protein
SAAV_1015	2.68398	0.031657	hypothetical protein
SAAV_1044	4.726664	0.000758	Putative cell-wall binding lipoprotein
SAAV_1047	3.004325	0.0104	hypothetical protein
SAAV_1071	4.71402	0.000538	manganese transport protein MntH
SAAV_1142	2.330614	0.049167	hypothetical protein
SAAV_1144	2.298832	0.044331	anti protein (phenol soluble modulin)
SAAV_1145	2.766178	0.015271	anti protein (phenol soluble modulin)
SAAV_1186	3.084378	0.008651	hypothetical protein
SAAV_1344	4.816015	0.000756	ImpB/MucB/SamB family protein
SAAV_1467	3.400655	0.007701	hypothetical protein
SAAV_1478	4.194828	0.003177	hypothetical protein
SAAV_1616	2.521719	0.028585	luciferase family protein
SAAV_1732	3.14251	0.010857	OsmC/Ohr family protein
SAAV_1749	2.407528	0.039931	hypothetical protein

SAAV_1782	3.064723	0.013929	hypothetical protein
SAAV_1785	2.671456	0.037926	N-acetylmuramoyl-L-alanine amidase
SAAV_1792	4.987387	0.005556	hypothetical protein
SAAV_1858	2.845326	0.014416	hypothetical protein
SAAV_1864	2.555347	0.033772	hypothetical protein
SAAV_1916	3.155847	0.013189	multidrug ABC transporter ATP-binding protein
SAAV_1921	2.846614	0.023246	ABC transporter ATP-binding protein
SAAV_1922	2.929204	0.024231	hypothetical protein
SAAV_1926	4.732839	0.003207	hypothetical protein
SAAV_1927	2.737978	0.03243	hypothetical protein
SAAV_1945	2.864782	0.018883	hypothetical protein
SAAV_1946	2.477162	0.036493	phosphotyrosine protein phosphatase
SAAV_1948	2.5293	0.034532	ribonuclease BN, putative
SAAV_1951	3.248174	0.012886	hypothetical protein
SAAV_1984	2.935061	0.016389	hypothetical protein
SAAV_1989	3.300771	0.00866	hypothetical protein
SAAV_1990	5.648788	0.000253	hypothetical protein
SAAV_2012	25.02691	3.32E-10	hypothetical protein
SAAV_2036	105.3432	9.01E-08	hypothetical protein
SAAV_2037	5.066737	0.036181	hypothetical protein
SAAV_2038	5.086748	0.000927	hypothetical protein
SAAV_2039	29.84778	1.07E-10	dUTPase
SAAV_2043	3.530976	0.011697	hypothetical protein
SAAV_2199	2.371054	0.042383	hypothetical protein
SAAV_2207	4.09523	0.002255	ABC transporter ATP-binding protein
SAAV_2210	7.094827	9.32E-06	PTS system, mannitol-specific IIBC components
SAAV_2211	3.37416	0.003929	BglG family transcriptional antiterminator
SAAV_2212	2.84314	0.012529	PTS system, mannitol-specific IIA component
SAAV_2242	2.486671	0.029779	Siderophore biosynthesis protein
SAAV_2349	3.429034	0.011535	putative urea transporter
SAAV_2375	6.771505	5.76E-05	hypothetical protein
SAAV_2391	2.475795	0.036282	hypothetical protein
SAAV_2392	2.66645	0.020134	short chain dehydrogenase/reductase familyoxidoreductase
SAAV_2468	5.196631	0.000218	formate/nitrite transporter family protein
SAAV_2469	45.67934	6.79E-12	hypothetical protein
SAAV_2470	4.628604	0.005391	hypothetical protein
SAAV_2496	2.945814	0.027542	hypothetical protein
SAAV_2497	3.596474	0.006102	hypothetical protein
SAAV_2538	2.339963	0.044621	putative short-chain dehydrogenase
SAAV_2541	7.776673	1.06E-05	hypothetical protein

SAAV_2544	2.468584	0.035216	short chain dehydrogenase/reductase familyoxidoreductase
SAAV_2560	4.485208	0.037451	hypothetical protein
SAAV_2574	3.039133	0.012844	hypothetical protein
SAAV_2575	3.784821	0.002482	hypothetical protein
SAAV_2592	2.576608	0.041825	hypothetical protein
SAAV_2603	3.180738	0.011783	hypothetical protein
SAAV_2615	6.329061	0.002231	hypothetical protein
SAAV_2616	3.59795	0.007373	ferrous iron transport protein B
SAAV_2631	3.458262	0.005153	hypothetical protein
SAAV_2658	2.571757	0.034544	hypothetical protein
SAAV_2671	3.408668	0.004728	hypothetical protein
SAAV_2750	2.770928	0.040436	histidinol-phosphate aminotransferase, putative
SAAV_2769	6.334185	0.000108	hypothetical protein
sarA	2.706385	0.018333	accessory regulator A
sdrE	5.586339	0.000141	sdrE protein
ssb1	3.349047	0.011486	single-stranded DNA binding protein
thyA	2.344314	0.046559	thymidylate synthase
tpiA	5.477747	0.000123	triosephosphate isomerase
trpA	2.964729	0.031092	tryptophan synthase subunit alpha
trxA	2.869513	0.013116	thioredoxin
ureB	4.162383	0.002621	urease subunit beta
ureC	4.526971	0.000846	urease subunit alpha
ureD	3.345681	0.00663	urease accessory protein UreD
ureE	4.127082	0.002252	urease accessory protein UreE
ureF	4.047339	0.002263	urease accessory protein UreF
ureG	4.262262	0.001419	urease accessory protein UreG

Supplementary table S4. Primers used in RT-qPCR

Gene	Direction	Sequence (5’-3’)
blaZ	forward	ATACTTCAACGCCTGCTGCT
	reverse	ACCTTATAGTCTTTTGGAACACCA
eno	forward	CGTACAGCTGCAGAACAAGTTGA
	reverse	CACCGATACGTTCTGTAAGTTGTTTC
ermC	forward	CCATTAAAAATAATGCCAATGAGCG
	reverse	TTAATCGTGGAATACGGGTTTGC
icaA	forward	CTTGCTGGCGCAGTCAATAC
	reverse	GTAGCCAACGTCGACAACTG
nuc	forward	AGCGATTGATGGTGATACGG
	reverse	TTGCTTCAGGGCCATATTTC
SAAV_1916	forward	TCGTCCAACAGCAACAGATGA

	reverse	TCATATCCCTGTGGCAAGTTCA
sarA	forward	GTAATGAGCATGATGAAAGAACTGT
	reverse	CGTTGTTTGCTTCAGTGATTCG
sdrE	forward	CAAAACCAACTACACCTCAAGAATCTA
	reverse	CTGGTTCTTTTGGATTAGTTGCATCT
thyA	forward	CTTCCGCCTTGTCATACCAT
	reverse	GCGATATTAAATGGCACACC
ureF	forward	GGACAAACTGCTGGTCAAAAGAT
	reverse	CATGCGCCATTTGATTTAGTTC

Supplementary Figures

Supplementary figure S1. Heat map of differentially expressed genes in MRSA1679a and ATCC 29213. Each column stands for one strain and each row represents a gene. Red color represents up-regulation while green color shows down-regulation of genes. Scale in the upper segment represents the calculated log2 fold change.

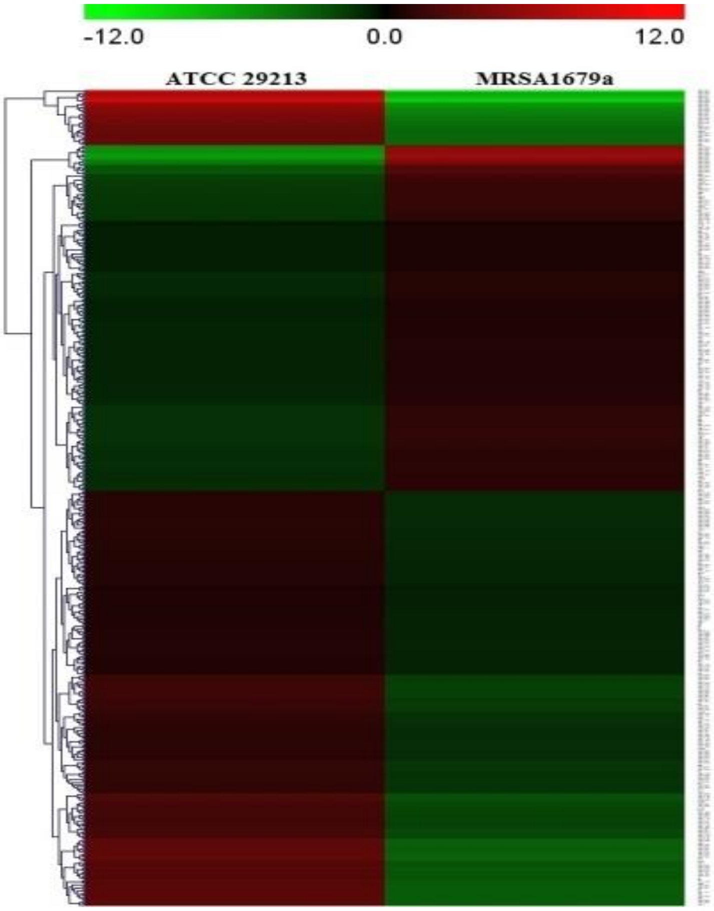

Supplementary figure S2. Differential expression ratio (log2) achieved by RT-qPCR and RNA-seq for the selected differential genes.

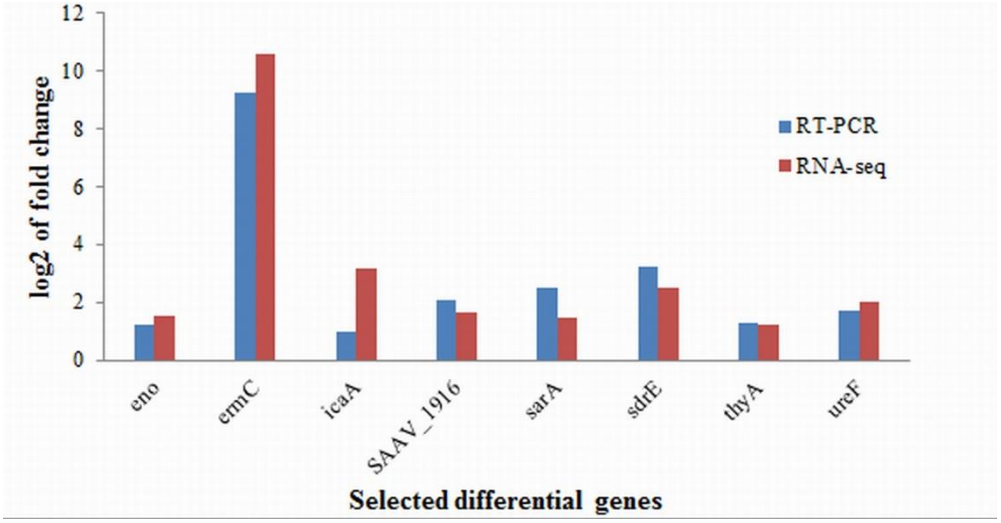

Supplementary figure S3. Correlation of differential expression ratio (log2) achieved by RT-qPCR and RNA-seq for the selected differential genes.

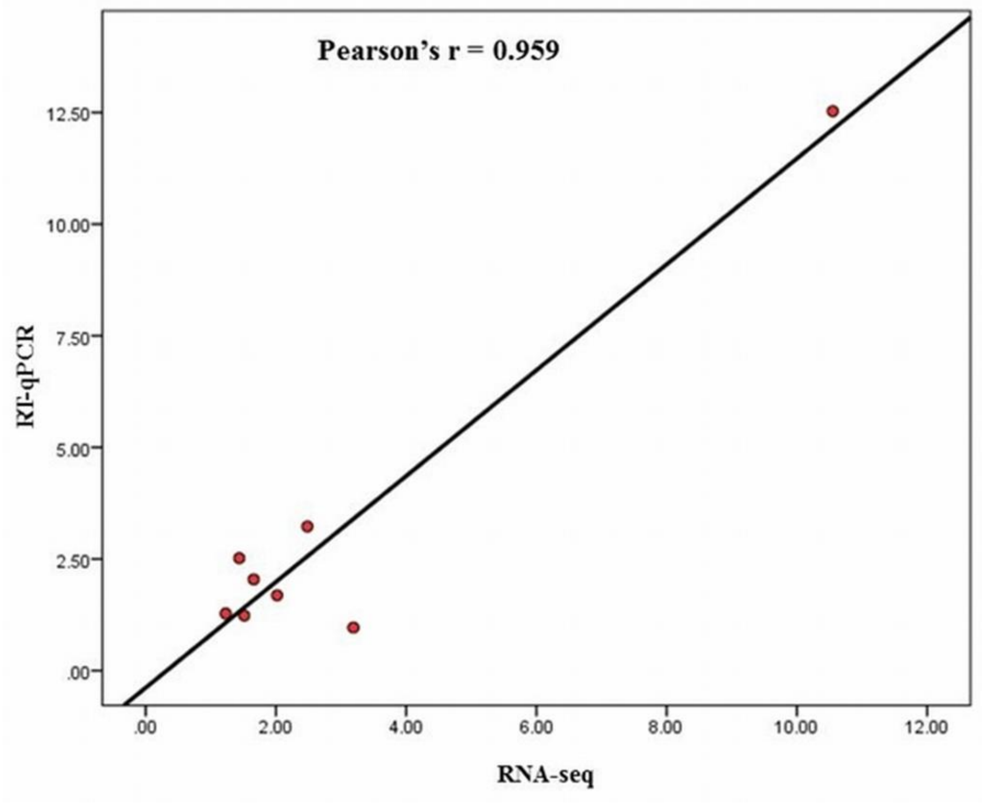

Supplement: Supplementary Information [file srep35442-s1.pdf]
